# Comparative Outcomes of Primary Probing Versus Lacrimal Sac Massage in Older Infants With Congenital Nasolacrimal Duct Obstruction

**DOI:** 10.7759/cureus.98945

**Published:** 2025-12-11

**Authors:** Lakshmi K Sreedharamurthy, Deepti Joshi, Krishnaprasad Ramakrishna

**Affiliations:** 1 Ophthalmology, Subbaiah Institute of Medical Sciences, Shivamogga, IND; 2 Ophthalmology, M M Joshi Eye Institute, Hubballi, IND

**Keywords:** cnldo, congenital nasolacrimal duct obstruction, epiphora, infants, lacrimal apparatus disease, lacrimal sac massage, probing

## Abstract

Introduction

Congenital nasolacrimal duct obstruction (CNLDO) is one of the most common causes of persistent epiphora and discharge in infants. While lacrimal sac massage is frequently advised as an initial therapy, the role of early probing in infants older than six months remains a subject of discussion. This study aimed to compare the outcomes of primary probing and lacrimal sac massage in infants aged 6-12 months with CNLDO.

Methods

A prospective interventional study (non-randomized controlled trial) was conducted over a one-year period involving infants aged 6 to 12 months diagnosed with CNLDO. Participants were assigned to two groups: Group 1 underwent primary probing, while Group 2 was instructed to perform lacrimal sac massage for three months. Treatment success was defined as the absence of clinical signs of CNLDO, namely watering, increased tear lake, and mucoid discharge. Analysis of data was performed using IBM Corp. Released 2017. IBM SPSS Statistics for Windows, Version 23. Armonk, NY: IBM Corp.

Results

A total of 128 infants were included; 48 children with CNLDO underwent primary probing, and 80 were treated with lacrimal sac massage. Probing achieved a significantly higher success rate (83.33%) compared to massage (25%). A chi-square test comparing the difference in success rates was highly statistically significant (p < 0.0001). Identified contributors to massage failure included poor compliance, improper technique, and recurrent upper respiratory infections.

Conclusion

Based on the significantly superior success rates and favorable compliance observed in our patient cohort, our findings suggest that early referral to an ophthalmologist and a decision for primary probing may represent a more efficacious initial treatment strategy than prolonged massage in this specific age group. Further larger-scale, multi-center trials are warranted to confirm these findings and provide a comprehensive assessment of long-term complication rates for both modalities.

## Introduction

Congenital nasolacrimal duct obstruction (CNLDO) affects approximately 6% to 20% of infants and is among the most common ophthalmic conditions encountered in pediatric practice [1,2]. Standard early management typically includes hydrostatic lacrimal sac massage combined with topical antibiotics. Many clinicians believe that a majority of obstructions resolve spontaneously with massage; however, around 25% of children may require additional intervention such as probing [3]. Currently, there is no established consensus on the optimal timing for referral to a pediatric ophthalmologist for probing. Because of the high spontaneous resolution rates documented in early infancy, some authors recommend postponing probing until after one year of age [2,3]. Conversely, other studies suggest that delaying probing beyond six months may increase the risk of inflammation and fibrosis, potentially lowering the success rates of later interventions [4,5]. This has resulted in a grey zone regarding the appropriate age for probing, generally considered to be between 6 and 15 months. The purpose of this study is to compare the outcomes of primary lacrimal sac massage versus primary probing for CNLDO in infants aged 6 to 12 months, with the goal of recommending an evidence-based timeframe for ophthalmologic referral and intervention.

## Materials and methods

We studied 128 children with CNLDO. The age range in both groups was between 6 and 12 months. The patients were divided into two groups. The group selection was on the basis of parents' choice at the time of diagnosis. The first group consisted of 48 infants in whom NLD probing was chosen as the primary treatment measure. The second group consisted of 80 infants who were advised to have lacrimal sac massaging as primary treatment.

Patients undergoing both unilateral and bilateral probing were included in the study. Other possible causes of epiphora, such as ectropion and allergy, were ruled out by careful examination of the anterior segment prior to probing. Probing was performed under brief general anesthesia with a Bowman lacrimal probe through both the upper and lower puncta following dilation with a Nettleship punctual dilator. After probing, the patients were placed on antibiotic-steroid combination eye drops, four times a day, for 10 days. They were also advised to do Crigler massage of the lacrimal sac area, 10 strokes for four times a day post-surgery. They were observed for resolution during the next month. Patency was verified in children after four weeks. Patency verification was done through a series of tests, which included clinical examination, the ROPLAS test, tear meniscus height, and lacrimal punctum examination for the presence or absence of discharge. In doubtful cases, a fluorescein dye disappearance test was performed.

Patients of group 2 were treated with lacrimal sac compression and massage with antibiotic eye drops. The correct method of lacrimal massage with occlusion of the common canaliculus and firm downward pressure on the lacrimal sac, using demonstration and an instructional video, was taught to the parents or guardians accompanying the patients, and they were instructed to properly perform 10 strokes four times a day. They were observed for spontaneous resolution during the next three months with patency verification tests every month.

The difference in follow-up periods reflects the nature of the treatments: Probing (Group 1) is an immediate mechanical procedure where success is evident within a month, justifying a shorter one-month check; conversely, lacrimal sac massage (Group 2) is a slow hydrostatic process requiring a prolonged observation of three months to allow the treatment to fully resolve the obstruction.

The outcome was labeled 'S' (Successful)... and 'F' (Failure) if any test suggested incomplete resolution in each patient, and the data was statistically analyzed. Probable causes of failure were tabulated for each case, and the data were analyzed by IBM Corp. Released 2019. IBM SPSS Statistics for Windows, Version 25. Armonk, NY: IBM Corp.

## Results

A total of 128 children with CNLDO were enrolled in the study. Table 1 outlines the baseline characteristics of the study participants, separated by treatment group. Group 1 (primary probing) included 48 children, and Group 2 (lacrimal sac massage) included 80 children.

**Table 1:** Baseline Demographic Characteristics of Children with CNLDO by Treatment Group CNLDO: Congenital Nasolacrimal Duct Obstruction

Group	Intervention done	Sample size (N)	Average age (in months)
Group 1	Primary Probing	48	9.1
Group 2	Lacrimal Sac Massage	80	8.9

The mean age of the patients was similar between the two groups: 9.1 months (Group 1) and 8.9 months (Group 2), confirming that the age distribution was comparable at the time of enrollment. The age range for all participants was consistently between 6 and 12 months. Gender distribution was also comparable, with 52.1% of patients in Group 1 being male and 55.0% in Group 2. 

Of the 128 children who had CNLDO, 48 (37.5%) children with CNLDO who underwent primary probing had a success rate of 83.3%, and 80 (62.5%) children who were treated with lacrimal sac massage as primary treatment had a success rate of 25%. There were no intraoperative complications related to GA or the probing procedures that were encountered in our study subjects. Data was analyzed using IBM Corp. Released 2019. IBM SPSS Statistics for Windows, Version 25. Armonk, NY: IBM Corp. Statistical analysis was done using the chi-square test for two proportions.

Since the p-value is << 0.05 as per Table 2 is extremely low, this confirms that primary probing is significantly more successful than lacrimal sac massage for CNLDO, with the difference unlikely to have occurred by chance. 

**Table 2:** Comparison of Primary Probing and Lacrimal Sac Massage for CNLDO CNLDO: Congenital Nasolacrimal Duct Obstruction, Data are presented as N (%)

Treatment	Success	Failure	Total	χ2 value	Sig (p-value)
Probing	40 (83.33%)	8 (16.67%)	48 (100%)	40.99	<0.0001
Massage	20 (25%)	60 (75%)	80 (100%)		
Total	60	68	=Highly Statistically Significant		

The causes of failure differed significantly between the two treatment groups, as detailed in Table 3. 

**Table 3:** Analysis of Causes of Treatment Failure by Group p-values are calculated using the Fisher's exact test comparing the distribution of causes between the two failure groups.

Causes of failure	Probing failure	Massage failure	P values	Significance
Structural/Anatomical Issues	7 (87.5%)	15 (25.0%)	<0.001	Statistically Significant
Non-Compliance/Technique	1 (12.5%)	20 (33.3%)	0.210	Not significant
Recurrent Infection (URI)	0 (0.0%)	25 (41.7%)	<0.001	Statistically Significant
Total Failures	8	60		

In the primary probing failure group (N=8), the overwhelming majority of failures were attributed to structural/anatomical issues (7/8, 87.5%), which suggests failure was primarily due to a resistant underlying pathology. Conversely, factors like recurrent infection were not noted as a cause of probing failure in our small subgroup.

In the lacrimal sac massage failure group (N=60), the reasons for failure were more distributed, with recurrent infection (URI) being the single most common cause (25/60, 41.7%). This cause was statistically significant in driving lacrimal sac massage failure (p < 0.001). Non-compliance/technique was a substantial factor (20/60, 33.3%) in the massage failure group, although the difference in its frequency as a cause of failure between the two groups was not statistically significant (p=0.210).

Crucially, structural/anatomical issues were significantly less frequent in the massage failure group (25.0%) compared to the probing failure group (87.5%) (p < 0.001). This difference underscores the contrasting mechanisms of failure. Probing fails due to tough anatomical resistance, while massage fails primarily due to environmental and patient factors like infection and non-compliance.

## Discussion

Pediatricians are the first line of contact for most children with CNLDO in our country. In recent years, there has been a heightened discussion regarding the optimal care of a child with CNLDO. The most important controversial topic has always been the timing of referral of a child with CNLDO to an ophthalmologist.

The current protocol managed by our pediatricians is a referral at 6-9 months of age for probing [6-9]. A study by Bhandari et al. highlights a failure rate of 70% with lacrimal massage alone and the increase in the failure rate of probing by 25% for every six-month delay, and this made us think, is it worth waiting for probing for approximately a year or a year and a half (as practiced today) or performing a probing early at six months of age [7].

Hence, through this study, we compared the efficacy of probing versus massage in children of 6-12 months with CNLDO.

Sac massage remains the first line of management until six months of age, but beyond six months of age, the success rate for sac massage varies from 28 to 95% [6-8]. Our study showed 25% success with sac massage beyond six months of age. The monotony of sac massage, improper technique, recurrent upper respiratory tract infections, and underlying chronic inflammation of the nasolacrimal duct are a few causes that may significantly influence lower outcomes.

Probing has been a time-proven treatment for congenital nasolacrimal duct obstruction. But there is controversy regarding the timing of probing, making it a double-edged sword. Our study showed an 83% success rate with probing at six months, encouraging early probing, and this has been supported by various studies in the past, suggesting that early probing avoids months of morbidity due to watering and chronic dacryocystitis. It has also been emphasized that delayed probing may result in decreased success with just a simple probing because of chronic inflammation and secondary fibrosis [9-11]. Yet not many people follow it, as CNLDO is considered a spontaneously resolving pathology, and early probing has been negated by many authors [11,12].

Hence, we analyzed all the factors responsible for this debate: A) Does CNLDO resolve spontaneously?: Analyzing consecutive CT faces over a 16-month period, Moscato et al. showed that the increase in height, diameter, and volume of the nasolacrimal duct occurs primarily in the first six months of life, suggesting that spontaneous resolution of obstruction in infants may be linked to this anatomic evolution, in which case probing before the age of five months should be avoided [13]. Macewen and Young followed children with symptoms of CNLDO till one year of age and calculated the chances of spontaneous resolution, where they predicted a spontaneous recovery until six months of age, which then either plateaued or reduced further (and this has been further confirmed by Sathiamoorthi et al. [13]). B) Is general anesthesia safe as early as six months? A short inhalational anesthesia is needed for the probing procedure in infants 6-12 months. Current evidence from multiple clinical studies and authoritative reviews indicates that a single, short exposure to general anesthesia in infants aged 6 to 12 months is not associated with detrimental long-term neurodevelopmental or cognitive effects. Regulatory agencies such as the U.S. Food and Drug Administration (FDA) acknowledge that while prolonged or repeated exposures may carry risks, brief anesthetic procedures necessary for surgery or diagnostic interventions are considered safe when carefully administered by pediatric anesthesiologists under appropriate monitoring. This understanding supports the use of short-duration general anesthesia in infants requiring timely surgical treatments, balancing therapeutic benefits against minimal risks [14-17].

The present prospective study demonstrates that primary probing is significantly more effective than lacrimal sac massage for treating congenital nasolacrimal duct obstruction (CNLDO) in children aged 6-12 months. The success rates of 83.3% for probing and 25% for massage highlight a clear therapeutic advantage of early surgical intervention, supported by a statistically significant difference (p<0.0001).

Consistent with published literature, probing in infants under one year of age achieves success rates exceeding 80% irrespective of anesthesia modality, aligning with findings from recent meta-analyses and multi-center trials. Conversely, lacrimal sac massage shows highly variable outcomes and markedly reduced efficacy beyond six months of age, often attributable to factors such as caregiver non-compliance, improper technique, and recurrent upper respiratory infections (URIs). These challenges in massage adherence, well documented in previous reports, substantially limit its long-term utility [18,19].

Early probing not only reduces the duration of symptomatic epiphora and discharge but also mitigates risks associated with chronic inflammation and infection. The low complication rates observed corroborate prior studies endorsing probing as a safe and reliable first-line treatment for persistent CNLDO beyond the neonatal period. While conservative management remains appropriate for infants younger than six months due to the natural resolution potential, our data and those of similar investigations advocate for prompt ophthalmic referral and intervention with probing in children six months and older.

This study has several limitations that should be acknowledged. First, the sample size, although adequate for initial comparison, may limit the generalizability of the findings to broader populations. Second, variability in parental compliance and technique with lacrimal sac massage could have introduced bias affecting the observed success rates. Third, the study’s follow-up period focused on short-term outcomes; longer-term follow-up would be necessary to evaluate sustained success and late complications. Additionally, non-randomized allocation of treatment groups may introduce selection bias, and reliance on clinical signs for outcome assessment may introduce observer bias. A methodological limitation of our study is the difference in follow-up duration prior to final patency assessment, which was dictated by the nature of the interventions, thereby creating the risk of detection bias. Another limitation of our study is the lack of detailed analysis concerning socioeconomic status (SES), caregiver educational level, or specific environmental risk factors that may influence compliance and infection rates. While clinical observation suggests these factors, along with poor general health and recurrent URIs, may significantly influence the success of prolonged, home-administered therapies like lacrimal sac massage, their quantitative impact on treatment failure could not be determined. Despite these limitations, the findings provide valuable evidence supporting primary probing as a superior treatment in infants over six months of age with CNLDO. Future randomized controlled trials with larger cohorts and standardized protocols would further validate these results and clarify optimal management strategies.

## Conclusions

Lacrimal sac massage (LSM) remains the established initial treatment modality for congenital nasolacrimal duct obstruction (CNLDO) in infants younger than six months of age. However, the outcomes of prolonging conservative management, as demonstrated in our study, are significantly influenced by clinical factors such as the high incidence of recurrent upper respiratory infections (URIs) and overall poor general health observed in our local patient population, which contribute to high massage failure rates. This study supports current consensus recommendations that advocate for primary probing as the treatment of choice for CNLDO in infants six months and older. Given its statistically superior success rate (83.33% for probing vs. 25% for massage), minimal procedure-related complications, and reduced duration of morbidity, we recommend that all children over six months of age be promptly referred to an ophthalmologist for consideration of definitive probing to achieve rapid and reliable resolution of CNLDO.
